# Improved resolution of mixed STR profiles using a fully automated differential cell lysis/DNA extraction method

**DOI:** 10.1080/20961790.2019.1646479

**Published:** 2019-08-20

**Authors:** Matthew C. Goldstein, Jordan O. Cox, Lori B. Seman, Tracey Dawson Cruz

**Affiliations:** Department of Forensic Science, Virginia Commonwealth University, Richmond, Virginia, USA

**Keywords:** Forensic sciences, forensic genetics, sexual assault evidence, QIAcube, differential lysis, sperm lysis, automated DNA extraction, mixture analysis

## Abstract

Sexual assault evidence often contains sperm cells, which are typically separated from nonsperm cells using manual differential lysis procedures. The goal of this study was to evaluate the automated QIAGEN QIAcube for this purpose and to compare it to manual QIAGEN and manual organic differential methods using DNA yields *and* STR profile data for assessment. DNA yields were determined by qPCR, followed by multiplex STR amplification, CE analysis, and mixture interpretation. The automated method was capable of effective cell separation, producing DNA yields sufficient for STR amplification. Further, sperm fraction human:male DNA ratios from the QIAcube samples were consistently closer to the desired 1:1 and STR profiles were less likely to result in mixtures, with 6–8× fewer female alleles detected (median 1.5 alleles). Ultimately, using the QIAcube for automated differential processing of semen-containing mixtures reduces the need for downstream mixture interpretation and improves STR profile quality with substantially less hands-on time.

## Introduction

Organic extraction and manual solid phase extraction are common forms of DNA extraction methods historically used in forensic DNA laboratories [1]. However, solid phase methods offer several advantages, including use of non-hazardous chemicals, fewer tube-to-tube transfers, less hands-on time, and opportunity for automation without the need for advanced manual pipetting [2–11]. Unfortunately, when sperm cells are expected to be present in a sample, such as those submitted with sexual assault cases, a differential lysis procedure is needed in order to differentially lyse, then separate sperm from nonsperm cells [2]. Even when back-end DNA extraction is automated, the differential lysis process includes an up-front manual removal of the aqueous epithelial cellular debris layer, which is labour intensive and skill-dependent, leading to varying results based on the experience of the examiner. These issues can result in lack of reproducibility, inefficient cell separation, and delayed processing—which can contribute to lagging backlogs. Several rapid and/or automated methods for differential separation of sperm cells have been developed as faster alternatives, including the use of alkaline plate-based methods [12], laser microdissection [13, 14], antibody-based cell capture [15, 16] and microdevice-based methods [17–19]; unfortunately, these techniques have not been widely adopted as they are either too laborious and complex or are not yet commercially available.

Many forensic laboratories have employed small scale robotic systems such as the EZ1 Advanced XL (QIAGEN, Valencia, CA, USA) and the Applied Biosystems Automate Express™ (ThermoFisher Scientific, Waltham, MA, USA) to improve the speed and reproducibility of cell lysis and DNA purification [6, 7, 20, 21]. While many of these systems are capable of lysing intact sperm heads, they do not currently possess the capability to physically separate sperm and nonsperm fractions on-deck (differential cell separation) [6, 7, 20, 21]. However, QIAGEN recently introduced a differential script for its QIAcube system, an automated sample preparation liquid handling instrument designed to process QIAGEN spin columns. The differential script allows for a robotic, on-deck processing of biological evidence samples, and includes initial cell lysis, separation of sperm/nonsperm fractions, and DNA purification. The responsibilities of the user simply include loading the plasticware, the chemicals, and the samples [22]. The QIAcube has been successfully used to extract DNA from a variety of starting evidentiary material, including bone, swabs, blood stains, and FTA cards [9, 23–26]. However, to date, there have been fewer publications which detail the performance of the QIAcube for differential forensic cell lysis and DNA extraction. In a lone study comparing seven DNA extraction protocols for separation of male and female contributor DNA, QIAcube was the only automated procedure tested, and it outperformed all but one of the other methods in both sperm fraction male DNA recovery and in improvements to female:male DNA ratios obtained [27]. Unfortunately, this study did not include a comparison of the QIAcube to more traditional differential lysis/DNA extraction protocols that continue to be predominant in forensic laboratories. Further, in these studies, qPCR-based quantitation results were singularly used as a basis for assessment of separation capabilities rather than capillary electrophoresis-based STR profile analysis. Thus, the goal of this study was to more fully evaluate the QIAcube as a method for forensic differential lysis and cell separation and to compare this method to existing, commonly used manual differential protocols.

## Materials and methods

### Sample collection and mixture preparation

Eleven female volunteers provided four buccal swabs each; buccal swab samples were used as the source of epithelial cells for the mock sexual assault samples produced for this study. Three swabs were used for experimental analysis while the fourth swab was used as a reference DNA sample. Additionally, four male volunteers provided semen for this study and two buccal swabs (used as reference DNA samples). All samples used in this study were collected in accordance with a university-approved Institutional Review Board protocol. To generate mock sexual assault samples, 3 µL of neat semen from a single male volunteer were added to each female dried buccal swab sample; semen from the four male samples were used as evenly as possible across each set of 11 buccal swabs. Care was taken to ensure each semen sample was adequately mixed before each pipetting repetition. One set of 11 epithelial cell-semen mixture swabs and one reagent blank were processed following each of the three methods below. In each of the three methods, the entire experimental swab was used for processing.

### Cell lysis and DNA extraction

One set of 11 swabs along with one extraction reagent blank were processed using the QIAcube automated sample prep platform (QIAGEN) following manufacturer’s recommendations for differential separation and DNA extraction, using standard QIAamp^®^ DNA Investigator Kit reagents (QIAGEN). Initially, epithelial cells were lysed using the “Buccal swab spin protocol part A (lysis)” program. Next, the sperm fraction and nonsperm fraction were separated and sperm cells were lysed using the “Differential wash protocol”. Both the sperm and nonsperm fractions were subsequently purified on-deck using the protocol “Buccal swab spin protocol part B (purification)”. An elution volume of 60 µL was used for both fractions.

A second set of 11 mixed buccal-semen swabs and one extraction reagent blank were extracted using a manual differential QIAGEN extraction method. Biological material from each swab was initially lysed with 400 µL of stain extraction buffer (1 mol/L Tris–HCl, ddH_2_O, 5 mol/L NaCl, 0.5 mol/L EDTA, 10% SDS, pH =8.0) and 15 µL of proteinase K (20 mg/mL) (Fisher Scientific, Pittsburgh, PA, USA) followed by an overnight 56°C incubation on a shaking platform. DNA IQ™ spin baskets (Promega Corporation, Madison, WI, USA) were then used to isolate the lysate from the solid material using a 7 500 × *g* spin for 5 min. The supernatant was transferred to a new tube and processed as the “nonsperm” fraction. The sperm pellet (contained within the original tube) was subsequently resuspended in 200 µL of phosphate buffered saline solution (Fisher Scientific), 20 µL of QIAGEN proteinase K stock solution, and 20 µL of 1 mol/L DTT (Fisher Scientific). After vortexing, 200 µL of Buffer AL (QIAGEN) was added, and samples were incubated at 56 °C on a shaking platform for 2 h. Following sperm cell lysis, both sperm and nonsperm fractions were purified manually using the QIAamp^®^ DNA Investigator kit following the “Isolation of total DNA from surface and buccal swabs” protocol per manufacturer’s guidelines. DNA was eluted in final volumes of 100 µL (nonsperm fractions) or 60 µL (sperm fractions) of Buffer ATE (QIAGEN).

A final set of 11 mixed buccal-semen swabs and one reagent blank were extracted using a traditional manual organic differential extraction method. The sperm lysis step was carried out in exactly the same way as the manual QIAGEN extraction method, with one exception. For organic extractions, 15 µL of proteinase K (20 mg/mL) (Fisher Scientific) was used along with the DTT for sperm fraction lysis. Following cell lysis, both sperm and nonsperm fractions were purified using an organic extraction method; for this, 500 µL of a phenol:chloroform:isoamyl alcohol mixture (25:24:1) (Sigma Aldrich Company LLC., Milwaukee, WI, USA) was added to each lysate then spun for 5 min at 18 400 × *g*; this was repeated once, for a total of two organic washes. The aqueous layer from each sample was then transferred to a pre-saturated (100 µL of TE Buffer (1 mol/L Tris–HCl, 0.5 mol/L EDTA, ddH_2_O)) Microcon^®^ Y-100 DNA Fast Flow Centrifugal Filter (Fisher Scientific) and spun at 350 × *g* for 13 min. The filtrate was discarded, an additional 200 µL of TE buffer was added, and each filter unit was spun at 350 × *g* spin for 18 min. Next, 75 µL of TE buffer were added to each filter unit and allowed to incubate at room temperature for 5 min. Finally, the filter units were inverted into new microcentrifuge tubes and spun at 950 × *g* for 5 min.

One buccal swab from each volunteer was also processed separately in order to develop a known reference DNA profile. Reference DNA was extracted with the QIAamp^®^ DNA Blood Mini Kit (QIAGEN) on the QIAcube following manufacturer’s protocol.

### DNA analysis methods

All DNA extracts were quantified using the Investigator^®^ Quantiplex HYres Kit (QIAGEN) with the ABI PRISM^®^ 7500 Sequence Detection System (Life Technologies™, Carlsbad, CA, USA) and SDS software v1.4.0 (Life Technologies™) following manufacturer’s protocol, with one modification (half reaction volumes were used). From the DNA concentrations provided (both the human and male targets), total human DNA yields and male DNA yields were determined by multiplying the concentration by the total original extract volume. Samples that were undetected were not reported in the resulting data; the human target failed to detect in a single sample from the manual organic set, and the male target failed to detect in a single sample from the manual QIAGEN set. Yields obtained using each method were compared using one-way ANOVA, followed by *post hoc* Tukey tests when applicable (α = 0.05). Human:male DNA ratios were calculated by dividing the total human yield by the total male yield. The two samples with the highest concentration, two samples with the lowest concentration and the two median samples from the QIAcube sample set, as well as those from the manual QIAGEN samples and those from the manual organic samples, were amplified using the AmpFℓSTR^®^ Identifiler^®^ PCR Amplification Kit (Life Technologies™) with a GeneAmp^®^ 9600 PCR System (PerkinElmer Incorporation, Waltham, MA, USA). Thermocycling conditions included a pre-denature step at 94 °C for 11 min, followed by 28 cycles of: denature 94 °C for 1 min, anneal 59 °C for 1 min, extension 72 °C for 1 min, and final post-extension step of 60 °C for 90 min. Amplification reactions each consisted of 5.7 µL of AmpFℓSTR™ PCR reaction mix, 2.0 µL of AmpFℓSTR^™^ Identifiler^™^ primer set, 0.20 µL of AmpliTaq Gold^®^ DNA polymerase, 2.1 µL of TE buffer, and 5 µL of 0.2 ng/µL template DNA (1 ng total).

All STR-amplified samples were separated by capillary electrophoresis (CE) on an Applied Biosystems^®^ 3130 Genetic Analyzer (ThermoFisher Scientific) using Data Collection software version 3.1 (ThermoFisher Scientific). For this step, 1.2 µL of amplified sample was added to 12.0 µL Hi-Di^™^ formamide (ThermoFisher Scientific), and 0.1µL GeneScan^™^ 500 LIZ^™^ size standard (ThermoFisher Scientific). CE run conditions followed the manufacturer’s recommendations and included use of POP-4^®^ polymer (ThermoFisher Scientific), a 36 cm capillary array (ThermoFisher Scientific), and a 10 s 3 kV injection. Electropherograms generated were analyzed using GeneMapper^®^ v4.1 software (ThermoFisher Scientific) with an analytical peak height threshold of 75 rfu. STR data from each sample were compared to the known reference STR profiles. For each sperm fraction analyzed, the number of alleles observed that could be attributed to the female donor were counted and a median was calculated for each group. Additionally, the number of sperm fraction samples that resulted in mixed profiles was counted; samples were deemed mixtures if one (in cases of homozygosity) or both (in cases of heterozygosity) known unshared expected alleles from the female contributor were detected in at least one STR locus.

## Results and discussion

**Figure 1.**
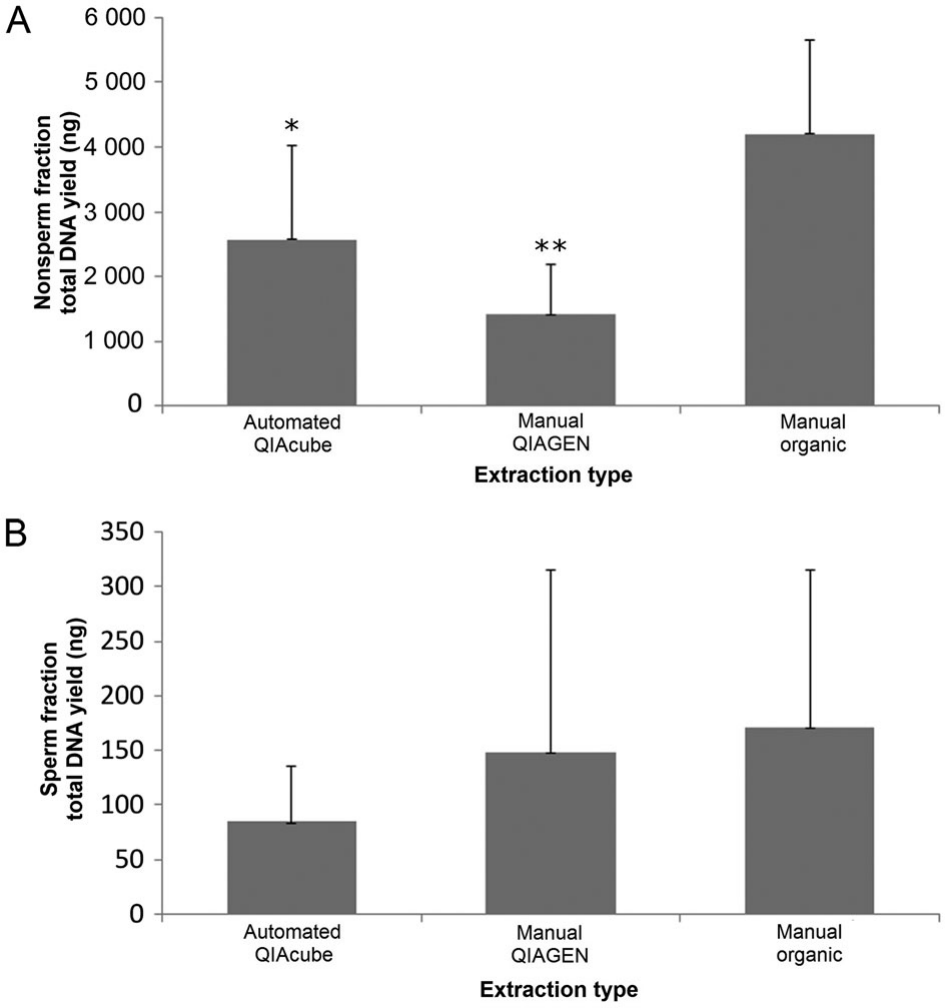
Total DNA yields from three differential cell lysis/DNA extraction methods. (A) The automated QIAcube method produced higher nonsperm fraction average DNA yields than the manual QIAGEN method, but this was not statistically significant (*P* = 0.09). The traditional manual organic method produced higher yields than both the automated QIAcube (*P = 0.02) and the manual QIAGEN (**P = 5.8 × 10^−5^) methods. (B) None of the three methods tested produced sperm fraction DNA yields that were statistically different when compared to each other (*P* = 0.29). *n* = 11 for automated QIAcube and manual QIAGEN, *n* = 10 for manual organic.

The primary goal of this work was to compare the separation and extraction capabilities of an automated differential lysis and cell separation technique (QIAcube) to that of two widely-used manual techniques using both quantitation and STR data for assessment. In this study, the automated QIAcube method resulted in higher, yet statistically insignificant DNA yields for the nonsperm fractions than the manual differential QIAGEN extraction method (*P* = 0.09), but lower yields than the manual organic differential extraction method (*P =* 0.02, Figure 1(A)). The traditional manual organic method also produced higher yields than the manual QIAGEN (*P =* 5.8 × 10^−5^) method. However, for sperm fractions, none of the three extraction methods provided significantly different total DNA yields when compared to each other (Figure 1(B), *P* = 0.29). It should be noted that regardless of the statistical significance, all methods tested resulted in yields that were in excess of 75 ng, on average, which is well beyond what is needed to develop a full STR profile [28–31].

**Figure 2.**
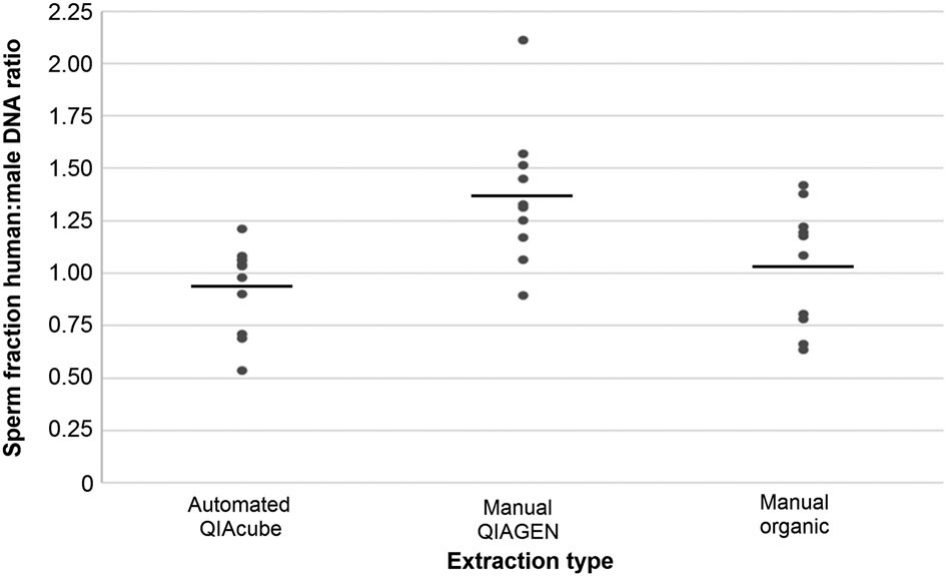
Sperm fraction total human:male DNA ratios from three differential cell lysis/DNA extraction methods. The manual QIAGEN method consistently resulted in higher human:male DNA ratios, indicating a more substantial female contribution remaining in the sperm fraction for this method and better separation for samples processed using the automated QIAcube and manual organic methods. *n* = 11 for automated QIAcube, *n* = 10 for manual QIAGEN and manual organic.

More importantly, when total human:male DNA ratios were evaluated for the sperm fractions, the automated QIAcube and manual organic methods were found to consistently result in ratios closer to the desired 1:1 than the samples processed using the manual QIAGEN method (mean ratios of 0.94:1 and 1.04:1 *vs*. 1.37:1, respectively) (Figure 2). Further, none of the sperm fraction samples processed using the automated QIAcube method produced quantitation values where the total human DNA content was greater than 1.2 times more than the estimated male DNA content, whereas 72% and 30% of sperm fraction samples processed using the manual QIAGEN method and the manual organic method, respectively, produced values that indicated a substantial female contribution (>1.2:1 human:male ratios). This is important because evidence samples that result in human:male ratios greater than 1:1 are more likely to result in a mixed STR profile [32–35], which requires a more tedious and time-consuming interpretation process than that which is commonly needed for single source profile interpretation.

**Figure 3.**
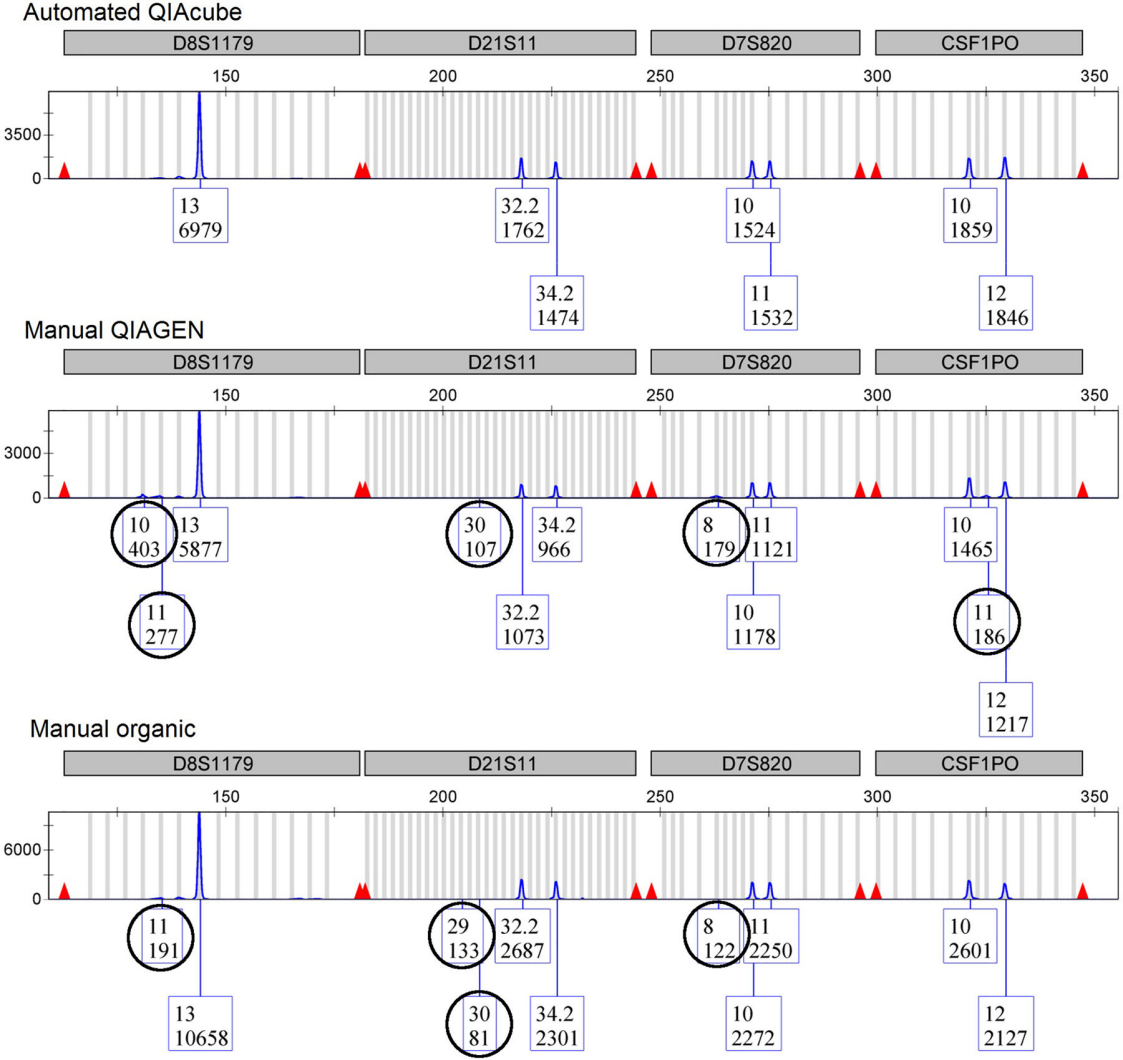
Representative sperm fraction electropherograms (blue channel) from three differential cell lysis/DNA extraction methods. (A) The automated QIAcube method is the only differential cell lysis/extraction method that resulted in a single-source STR profile for the sample shown. An increase in the number of female donor alleles was observed when the same mixture sample was processed using the manual QIAGEN method (B) or the manual organic method (C). STR alleles attributed to the female donor are circled.

The findings reported above from the quantitation data are further supported by the STR data obtained. Only two of six sperm fraction samples resulted in mixture STR profiles after processing with the automated differential QIAcube method versus five of six sperm fraction samples from each of the manual methods tested (Table 1). Additionally, in the two sperm fraction mixtures resulting from automated differential processing, the female contributor constituted an average of only 3.1% of the total DNA mixture. It is reported in the forensic DNA community that when a minor contributor is less than 5% of a two-person mixture (<20:1 major:minor), it is unlikely to impact analysis and consequently, the major contributor is likely easily interpreted as a single source profile [36]. Further, the data from this study show that the median number of residual female alleles detected in the sperm fraction electropherograms was 6–8× higher when manual methods were used (Table 1, Figure 3). Similar trends were noted in the STR data obtained from the nonsperm fraction samples, where fewer mixtures and fewer male alleles were observed when samples were processed using the automated differential method (data not shown).

**Table 1.** STR profile data from sperm fractions tested.

Method	No. of mixtures detected	Median number of female STR alleles
Automated QIAcube	2/6	1.5
Manual QIAGEN	5/6	12
Manual organic	5/6	9

## Conclusion

The goal of this study was to evaluate the “Differential Wash Protocol” for differential cell lysis and separation of forensic cell mixtures using the QIAGEN QIAcube automated sample preparation system. Few studies have evaluated the QIAcube’s ability to efficiently process semen-containing biological mixtures, which require a differential cell lysis. In this study, the automated QIAcube cell separation and lysis method was shown to be capable of differentially lysing and separating cells from semen-containing mixtures (using 3 µL of semen). DNA yields produced were comparable to commonly used manual methods and were suitable for downstream STR amplification. Moreover, total human:male DNA ratios suggested that the automated QIAcube method is more effective than the equivalent manual methods at separating nonsperm cells away from sperm cells. This observation was supported by the STR profile data generated, which showed that the automated method reduced the number of mixtures observed during STR profile analysis, producing profiles with negligible contributions from the female DNA. Most importantly, the hands-on time needed to achieve this improved cell separation and profile quality was reduced by at least 90 min, requiring only half of the time needed for the traditional manual differential methods tested. Future studies will address the sensitivity of this automated differential method.
